# Anticoagulation failure in pulmonary thromboembolism in COVID-19 pneumonia despite prolonged anticoagulation: A case series

**DOI:** 10.1016/j.amsu.2022.104929

**Published:** 2022-11-19

**Authors:** Prakash Sapkota, Ashish Tamang, Sadikshya Bhandari, Yadvinder Singh, Rohit Bhasink Shrestha, Robin Man Karmacharya, Satish Vaidya, Swechha Bhatt

**Affiliations:** a Department of Internal Medicine, Kathmandu University School of Medical Sciences, Dhulikhel Hospital, Dhulikhel, Kavre, Nepal; b Kathmandu University School of Medical Sciences, Dhulikhel Hospital, Dhulikhel, Kavre, Nepal; c Cardiothoracic and Vascular Surgery Unit, Department of Surgery, Kathmandu University School of Medical Sciences, Dhulikhel Hospital, Dhulikhel, Kavre, Nepal

**Keywords:** Anticoagulant, Case series, COVID-19, Pneumonia, Pulmonary thromboembolism

## Abstract

**Introduction:**

Moderate to severely ill patients diagnosed with Coronavirus disease 2019 (COVID-19) pneumonia develop a series of complications and less frequently, we might witness cases of Pulmonary Thromboembolism (PE)-refractory to the standard treatment with Low Molecular Weight Heparin (LMWH). The aim of this case series is to report the presentation and management of pulmonary thromboembolism secondary to COVID-19 pneumonia.

**Method:**

We report a case series of seven cases aged 40–70 who were presented in Dhulikhel Hospital with COVID-19 symptoms in different stages. The case details were extracted from their medical reports of the hospital. The written informed ethical consents were obtained from all the cases and their voluntary participation was assured.

**Outcome:**

The cases in the case series admitted with COVID-19 pneumonia, after diagnostic investigation (Chest x-ray, HRCT, CTPA) were suggestive of COVID-19 Pneumonia with ARDS and pulmonary thromboembolism. The cases received rivaroxaban, a newer anticoagulant-15 mg twice daily for 21 days and after discharge, they were asked to continue once daily doses for 9 weeks. Significant improvement was witnessed, with the presence of additional intervention including rehabilitative chest exercises.

**Conclusion:**

Pulmonary thromboembolism secondary to COVID-19 pneumonia is a life-threatening condition. Rivaroxaban is seen to be very effective in the management of this condition when an anticoagulation failure occurs even after the therapeutic dose of low molecular weight heparin. Future studies may require more scientific investigations to prevent complications even in the early stages of COVID-19.

## Highlights

• Seven patients with anticoagulation failure in Pulmonary Thromboembolism following COVID-19 pneumonia.
• Prolonged treatment of pulmonary thromboembolism for 3 months with novel anticoagulant-Rivaroxaban.
• Suggestion on individualized treatment protocol for risk stratification and preventing complications of COVID-19.

## 1 Introduction

Pulmonary thromboembolism (PE) is a hypercoagulative state caused by cytokine storm, hyperactivation of complement factors, and endothelial dysfunction [1]. PE in COVID-19 patients corresponds to nearly 0.5% of cases presenting to the Emergency Department [1]. However, the incidence is nine times higher when compared to PE in patients with no COVID-19 [1]. This hypercoagulable state has a poor prognosis and can be biochemically detected by increased D-dimer levels [1] (see Table 1, Table 2)

**Table 1:** Details of cases of COVID-19 pneumonia in the case series with PTE.

CASE	Sex	Age	Duration of hospital stay	Duration Of LMWH	Duration of rivaroxaban	Duration Of domiciliary oxygen	Repeated CTPA	Chronic Illness	BMI kg/m2
1	Male	42	23	21	3 months	3 months	Resolved	Hypertension	27.6
2	Male	60	28	21	3 months	3 months	Resolved	Hypertension	27.4
3	Male	53	21	21	3 months	2.5 months	Resolved	Diabetes	23.9
4	Male	70	16	14	3 months	2 months	Resolved	Parkinsonism Hypertension	26.2
5	Male	40	15	14	3 months	2 weeks	Resolved	Diabetes	22.4
6	Male	46	30	21	3 months	2 weeks	Resolved	Hypertension Diabetes Asthma	27.3
7	Male	60	30	14	3 months	3 months	Resolved	Hypertension	24.9

**Table 2:** Laboratory reports of Cases.

Parameters	Case 1	Case 2	Case 3	Case 4	Case 5	Case 6	Case 7	Reference Range
**Complete Blood Count ( × 10**^**3**^**/μL)**	4.7	65	7.9	8.5	10.9	10.9	47	4.0–11.0
**Neutrophil Count %**	78	85	92	88	93	86	78	45–75
**Lymphocyte Count %**	15	11	4	8	4	8	15	20–45
**Hemoglobin (gm/dl)**	14.3	12.5	13.2	13.3	15.1	13	14.3	12–16
**Platelets count ( × 10**^**3**^**/μL)**	271	317	162	135	196	148	150	150–450
**CRP Quantitative mg/L**	152	150	145	136	32	150	150	<5.0
**D- Dimer at the time of admission ng/ml**	3.8	1975	4	N/A	9.5	3.7	1975	<500
**ABG P02 mm of Hg**	54	58	48	40	58	62	53	80–100

Anticoagulant treatment with low molecular weight heparin (LMWH) should be considered in all hospitalized patients to prevent subsequent organ damage unless contraindicated [2]. It is suggested to improve indices of coagulation dysfunction on administration [3].

Here, we present seven cases of PE following moderate to severe COVID pneumonia despite the use of prolonged anticoagulation therapy. This article is written in line with PROCESS 2020 criteria [4].

## 2 CASE series

### 2.1 Case 1

A 42-year-old male presented to Dhulikhel Hospital with a complaint of acute onset fever with chills and rigor for three days. He also had shortness of breath (SOB) at rest for four days which was associated with a one-day history of dry cough, chest pain, and loss of taste. He had no complaints of orthopnea, paroxysmal nocturnal dyspnea (PND), palpitation, or loss of consciousness (LOC). He had no past diagnosis of asthma, chronic obstructive pulmonary disease (COPD), hypertension, or diabetes mellitus.

**Fig. 1:**
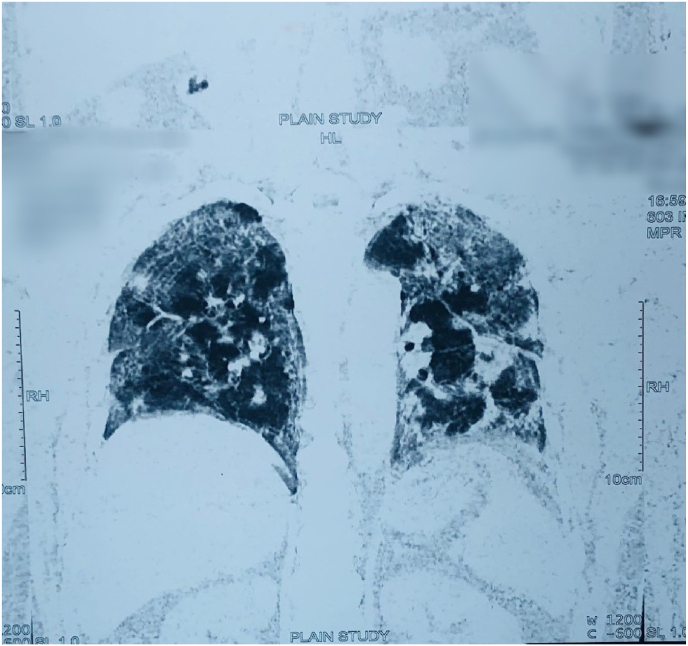
HRCT patchy ground-glass opacities in bilateral lungs with subpleural blebs.

On examination, his respiratory rate was 30 breaths per minute, and oxygen saturation (SpO2) was 92% with a 2 liter/min oxygen (O_2_) supply. The reverse transcriptase polymerase chain reaction (RT-PCR) test was positive for COVID-19 infection. As the patient was unstable and could not tolerate computed tomography pulmonary angiography (CTPA), we ordered a high-resolution computed tomography (HRCT**)** of the chest, which showed patchy ground-glass opacities in bilateral lungs with subpleural blebs (Fig. 1), suggestive of severe COVID-19 pneumonia. We put him on high-flow oxygen through a nasal cannula. Additionally, we added steroids, anticoagulants, and other medications such as paracetamol, fexofenadine, vitamin C and zinc, and rehabilitative chest exercise as a supplemental therapy to improve respiration. We also administered subcutaneous enoxaparin 40 mg twice daily continuously for 14 days, followed by a once-daily dose for 7 days.

**Fig. 2:**
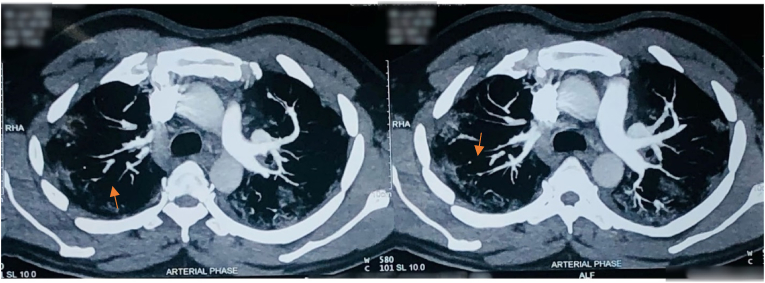
CTPA shows a contrast filling defect over the posterior basal segmental branch of the bilateral interlobar artery and anterior segmental branch of bilateral upper lobes as evidence of PE.

After the patient could tolerate low flow O_2_, we performed CTPA, which revealed a contrast filling defect over the posterior basal segmental branch of the bilateral interlobar artery and anterior segmental branch of bilateral upper lobes (Fig. 2), suggestive of pulmonary thromboembolism. (even after receiving prolonged anticoagulation)

Following this, we started the patient on rivaroxaban, a newer anticoagulant 15 mg twice daily over the first 21 days, followed by a dose of 20 mg once daily for the remaining 9 weeks. We then witnessed a significant improvement; thus, we discharged the patient on the 23rd day of admission with advice to continue rivaroxaban treatment for 9 weeks along with domiciliary oxygen and rehabilitative chest exercises.

**Fig. 3:**
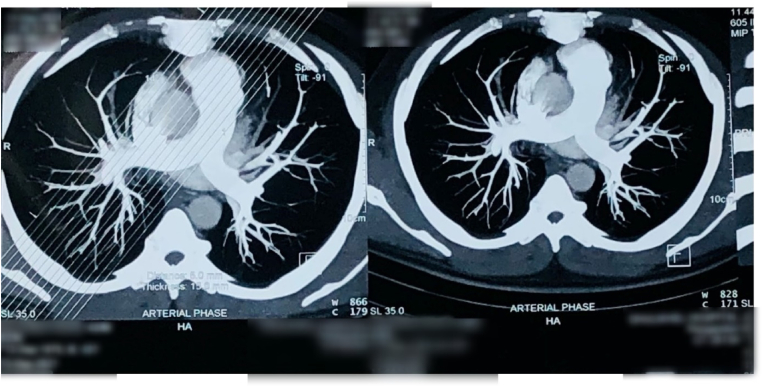
Follow-up CTPA showing resolution of previous contrast filling defect after rivaroxaban therapy.

The patient is on regular monthly follow-up, and CTPA performed following three months of discharge showed the resolution of the thrombus (Fig. 3).

### 2.2 Case 2

A 60-year-old hypertensive male presented to our center with complaints of acute onset, progressively increasing shortness of breath, and dry cough for a day. He had no orthopnea or PND, fever, headache, anosmia, or LOC. On examination, respiratory rate was 24 breaths per minute, and SpO2 was 95% at 4L/min O_2_ supplementation.

**Fig. 4:**
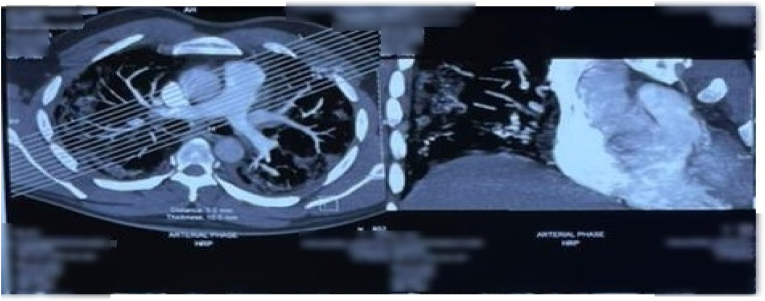
CTPA showing contrast filling defects over the posterior basal segmental branch of the bilateral interlobar artery, an anterior segmental branch of bilateral upper lobes, and in the mid basal segmental branch of the right interlobar artery.

The chest X-ray and HRCT-chest showed patchy ground-glass opacities in bilateral lungs with subpleural blebs with a computed tomography severity score of 16 out of 25 and CORADS score of 6. We placed him on high-flow oxygen through a nasal cannula and added steroids, anticoagulants, and other supportive medications. We also administered enoxaparin 40 mg subcutaneously twice daily for 14 days due to the high d-dimer value. The dose was gradually tapered. Even so, the patient deteriorated; thus, we shifted him to the COVID ICU and managed him with intermittent continuous positive airway pressure (CPAP). Once the patient was stable even on low flow oxygen, we ordered a CTPA, which showed contrast filling defects over the posterior basal segmental branch of the bilateral interlobar artery, an anterior segmental branch of bilateral upper lobes, and in the mid basal segmental branch of the right interlobar artery (Fig. 4). Therefore, we started the patient on rivaroxaban 15 mg twice daily for the first 21 days, followed by a dose of 20 mg once daily for 9 weeks. more months. We witnessed a significant improvement; hence, we discharged the patient on the 28th day of admission with advice to continue rivaroxaban for 9 weeks along with domiciliary oxygen and rehabilitative chest exercises.

The patient continued regular follow-up, and CTPA performed after three months showed the resolution of the thrombus.

### 2.3 Case 3

A 53-year-old diabetic male presented to the hospital with complaints of acute onset and progressive shortness of breath at rest for four days, and dry cough for three days, no orthopnea or PND, no history of fever, headache, or loss of consciousness. The respiratory rate was 24 breaths per minute on general examination, and SpO2 was 90% at 15L/min O_2_ supplementation.

We ordered a chest HRCT, revealing peripheral patchy ground-glass opacity and consolidations in bilateral lungs with secondary pulmonary lobular thickening and subpleural bands suggesting sequelae of COVID-19 pneumonia. The computed tomography severity score was 17 out of 25, and the CORADS score was 6.

For treatment, we administered steroids and anticoagulants along with insulin and other supportive medication, and he was placed on non-invasive ventilation. However, his d-dimer was increasing despite the use of anticoagulants, and thus, we continued him on subcutaneous enoxaparin 40 mg twice daily for 21 days, which was then changed to rivaroxaban. We also ordered CTPA, which showed eccentric contrast filling defect in the upper lobar and segmental branches of the pulmonary artery with a maximum of 50% luminal narrowing and eccentric contrast filling defect noted in the right descending branch of the pulmonary artery with less than 50% of luminal narrowing suggesting pulmonary thromboembolism.

We discharged the patient on the 21st day of admission with anti-diabetic medication. The patient was on a regular follow-up, and CTPA performed after three months showed the resolution of the thrombus.

### 2.4 Case 4

A 70-year-old male presented to the hospital with acute onset fever for three days, associated with chills. He also had severe shortness of breath for two days, even on rest, and a productive cough for the same duration. He had no hemoptysis, chest pain, orthopnea or PND. He is a known case of Parkinson's disease and hypertension under medication. The respiratory rate was 30 breaths per minute, and SpO2 was 89% in the 15 L/min oxygen supply.

We ordered an HRCT of the chest, which showed patchy and confluent ground-glass opacities in the peripheral parts of bilateral lungs with diffuse interlobular septal thickening, likely to be sequelae of COVID-19 pneumonia. The computed tomography severity score was 23 out of 25, and the CORADS score was 6.

We intermittently managed the patient with intermediate CPAP and bilevel positive airway pressure ventilation (BiPAP). We administered an injection of enoxaparin 40 mg subcutaneously once daily initially for ten days but then increased the dose to 60 mg subcutaneously twice daily for the next four days due to increasing levels of D-dimer and is due to his high Body Mass Index (BMI). We also gave him corticosteroids and other supportive medications. After the patient became stable in low oxygen, we ordered CTPA, which showed a contrast filling defect in the subsegmental branch of the lateral basal segmental branch of the left lower lobe suggestive of pulmonary thromboembolism. Thus, we started the patient on rivaroxaban 15 mg twice daily for the first 21 days, followed by a dose of 20 mg once daily for 9 weeks. He was discharged on the 16th day with medications. The case was on a regular monthly follow-up for about 3 months, and CTPA performed after three months showed thrombus resolution.

### 2.5 Case 5

**Fig. 5:**
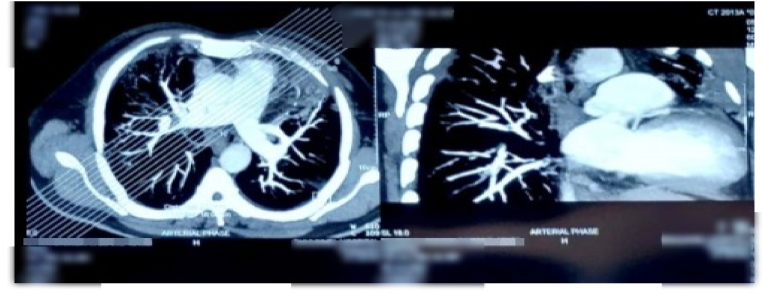
CTPA showing a contrast filling defect in the posterior basal segmental branch, a few subsegmental branches of bilateral interlobar pulmonary arteries, and the right upper lobe segmental branch.

A 42-year-old male presented with complaints of acute onset, progressive and severe SOB for four days which was associated with dry cough and acute onset fever for two days. He had no history of orthopnea, PND, chest pain, or palpitation. He had a history of bronchial asthma, hypertension, and diabetes mellitus and is under medication. His routine blood investigations showed a raised HbA1C of 7.6%, a raised fasting blood sugar of 207 mg/dl, and a raised post-prandial blood sugar of 436 mg/dl. The rest of the reports were within normal limits. We also ordered an HRCT of the chest, which showed peripheral patchy ground-glass opacities in bilateral lungs with segmental and subsegmental distribution suggestive of COVID pneumonia. His computed tomography score was 11 out of 25, and the CORADS category was 6.We kept him under ICU surveillance and placed him on intermittent CPAP and BiPAP. Anticoagulants, oral steroids, and subcutaneous injection of insulin with other supportive medications were given along with medication for asthma and hypertension. Once the patient was stable in low flow oxygen, we ordered CTPA, which showed a contrast filling defect in the posterior basal segmental branch, a few subsegmental branches of bilateral interlobar pulmonary arteries, and the right upper lobe segmental branch (Fig. 5).

Thus, we started the patient on rivaroxaban 15 mg twice daily for the first 21 days, followed by a dose of 20 mg once daily, which was continued for 9 weeks. We discharged the patient on the 15th day of admission with medications as given in prior cases and the advice to continue drugs prescribed for diabetes, asthma, and hypertension. The patient is on a regular follow-up, and CTPA performed after three months showed the resolution of the thrombus.

### 2.6 Case 6

A 40-year-old male complained of SOB persisting even at rest for three days along with dry cough for the same duration. He had no orthopnea or PND, no fever, headache, or LOC. His respiratory rate was 28 breaths per minute, and SpO2 was 90% at 15 L/min O_2_ supplementations. Blood evaluation showed an increase in random blood sugar level of 238 mg/dl with 6.3% of HbA1c.

HRCT was done, which showed multiple areas of ill-defined patchy ground-glass opacities noted in bilateral upper lobes, diffuse ground-glass opacities with air-bronchograms, and smooth interlobular septal thickening noted in bilateral lungs, predominantly in the perihilar location with cardiomegaly which was likely due to cardiogenic pulmonary edema. Echocardiography was done, which showed no regional wall motion abnormality.

Thus, we diagnosed the patient with severe COVID pneumonia and started him on injection of enoxaparin 60 mg subcutaneously twice daily for 14 days due to higher levels of d-dimer. We also gave him steroids with other supportive medications. He was placed on non-invasive ventilation with intermittent CPAP and BiPAP. Once the case was stable on low flow oxygen, we ordered CTPA and showed the contrast filling defect in the subsegmental branch of the posterior basal segment of bilateral pulmonary arteries, multiple areas of ill-defined patchy ground-glass opacities noted in bilateral upper lobes, and diffuse ground-glass opacities with air-bronchograms and smooth interlobular septal thickening and subpleural bands in bilateral lungs. It was a potential sequela of COVID pneumonia. Thus, we started the patient on rivaroxaban 15 mg twice daily for the first 21 days, followed by a dose of 20 mg once daily for 9 weeks more.

We discharged the patient on the 30th day of admission with the advice of medication as in prior cases. The patient is on a regular monthly follow-up, and CTPA performed after three months showed the resolution of the thrombus.

### 2.7 Case 7

A 60-year-old male with hypertension presented with acute onset fever for three associated with chills and rigors. He also had a sudden onset of progressively increasing SOB for the same duration, which was present even at rest, and was associated with dry cough and chest pain. He had no orthopnea or PND, no fever, headache, or LOC.

The chest HRCT showed patchy ground-glass opacities with interstitial and interlobular thickening and septal thickening in bilateral lungs with subpleural honeycombing and traction bronchiectasis. In the COVID care center, he received steroids, anticoagulants, and other supportive medication and high-flow oxygen via nasal cannula.

Once the patient improved clinically, we ordered a CTPA. It showed a contrast filling defect over the lingular segmental branch of the left pulmonary artery and the anterior segmental branch of the bilateral upper lobes. Additionally, there was an enlarged pulmonary artery and marked centrilobular and paraseptal emphysematous changes with diffuse interlobular septal thickening and fibrotic pulmonary bands. All the findings were suggestive of PE. Therefore, the case was treated using rivaroxaban 15 mg twice daily for the first 21 days, followed by a dose of 20 mg once daily for 9 weeks more. He got discharged on the 30th day of admission with the advice to continue the medication and the regular follow-up. The CTPA performed after three months showed the resolution of the thrombus.

## 3 Discussion

PE is a fatal condition usually associated with right ventricular dysfunction [5]. Many studies have suggested a correlation between COVID and increased thrombosis risk [1]. The incidence of pulmonary thrombosis in COVID-19-infected patients has been reported to be as high as 79% [6]. As PE and COVID-19 pneumonia share common signs and symptoms, differentiating PE in patients with COVID-19 is challenging [7]. Despite the use of therapeutic dose anticoagulation, 23% of the admitted COVID-19 patients disclosed pulmonary embolism [8]. PE engages multifactorial unified pathogenesis, which includes increased risk of thrombosis secondary to increased cytokines, endothelial injury, platelet activation, neutrophil traps, complement activity, hypoxia, and invasion of COVID-19 into the endothelial cells via angiotensin-converting enzyme 2 (ACE2) receptors as well as the presence of antiphospholipid antibodies[8].

PE is nine times more common in COVID-19 [1]. Apart from the common cause of thrombosis i.e. immobility and severe illness; studies have analyzed established cardiovascular risk factors in the development of VTE (Venous thromboembolism) in COVID-19 and concluded that adiposity, older age (68–75 years) and smoking were consistently associated with a higher risk of VTE where adiposity showed a stronger association in non-COVID-19 cases [9]. Other major unprovoked risk factors listed were hypertension, metabolic syndrome, air pollution, long-haul travel, and thrombophilias [10]. In the COVID-19 cases, the information on the associated factors in stratification of the risk factors in the patients to initiate the required prophylaxis. This case series presented older adult cases with at least one comorbidity and hypertension was the most common one. The series could not present other risk factors in detail.

Most of the signs and symptoms of PE and COVID-19 pneumonia are similar, therefore diagnosing PE in COVID-19 patients can be difficult and often lead to misdiagnosis, mistreatment, or a delay in treatment initiation, under-diagnosis of the condition may raise the risk of morbidity and mortality.7 These cases in this case series had confusing signs and symptoms similar to the findings of other studies.

In patients with positive COVID-19 infection, CT progression and increasing D-dimer levels are regarded as the most critical criteria suggesting underlying PE which is typically detected during the second week of infection and necessitates the use of CTPA to exclude or confirm PE [11]. The confirmation can provide an indication for the use of anticoagulation in either prophylactic or therapeutic doses [11]. The cases in the series underwent investigations similar to other studies such as CT, D-dimer, CTPA, and others, reflecting the need for the appropriate, timely, and precise diagnosis.

Several guidelines for both prophylaxis and treatment of thrombosis in hospitalized COVID-19 cases have been published. International Society of Thrombosis and Haemostasis (ISTH) interim guidelines recommend prophylactic dose low molecular weight heparin (LMWH) be considered in all patients, including non-critically ill, who require hospital admission for COVID-19 infection [2]. However, the CHEST guideline update published in the February of 2022 has made recommendations for acutely ill hospitalized patients with COVID-19 who have a low risk of bleeding to use therapeutic dose heparin (UFH or LMWH) over the current standard dose anticoagulant thromboprophylaxis [12]. However, in patients who are acutely ill but not receiving therapeutic doses of heparin, the current standard dose of anticoagulant thromboprophylaxis over intermediate-dose anticoagulation is recommended [12]. Moreover, in critically ill patients with COVID-19, current standard dose anticoagulant thromboprophylaxis (with UFH or LMWH) over both therapeutic dose anticoagulation and intermediate-dose anticoagulation is recommended [12]. As recommended by the guidelines, all the indexed cases in this case series received LMWH in therapeutic doses from the first day of hospital admission. After the stability under low flow oxygen, CTPA was done resulting in the diagnosis of PE in all the cases.

A study highlighted that among the cases of severe pneumonia and high BMI, the outcome of the prophylactic anticoagulation was poorer than the therapeutic anticoagulation, suggesting less effective thromboprophylaxis among cases with elevated BMI [14]. The cases in this case series reported failure in thromboprophylaxis and raised BMI levels (i.e. 24.9 kg/m^2^).

Some studies did not support the use of higher than standard doses for prophylactic anticoagulation in critically ill patients with COVID-19 due to the lack of immediately visible benefit and the risk of bleeding events. An experimental study conducted among 600 critically ill cases reported that the intermediate-dose anticoagulation with LMWH daily was not superior to standard prophylactic anticoagulation with LMWH daily in reducing the composite outcome of venous or arterial thrombosis [13]. The study also revealed that the bleeding events were rare but major and clinically relevant bleeding events were more frequent with intermediate-dose anticoagulation, albeit not significantly [13]. The indexed cases in this case series as well as other admitted cases during the COVID-19 pandemic also had no clinically relevant bleeding events or signs of pulmonary thrombosis even after therapeutic anticoagulation reflecting failure in thromboprophylaxis.

The cases in this case series received Vitamin C, following the evidence of increased recovery rate shown by reanalysis of the COVID A to Z Trial [14]. The chest exercises might improve respiratory functions enhancing the quality of life of the cases after their hospital stay, however, this has less evidence of enhancement in the COVID-19 treatment [17]. The cases presented in the case series adapted the chest exercise as the supplementary therapy, which added support to the cases in recovery and rehabilitation.

A study found that the group treated with glucocorticoids had an increased level of D-dimer than the control group. The heightened level of D-dimer elevates the risk of venous thromboembolism in COVID-19 patients by several folds [11]. This case series also presented cases with raised D-dimer levels and the occurrence of PE, despite using anticoagulants.

A multicentered clinical trial concluded that rivaroxaban monotherapy contributed to reducing the length of hospital stay, and decreasing the cost of treatment while being efficacious and well-tolerated in patients with PE [15]. In this case series, the cases were confirmed PE using CTPA, and then they were treated with the novel anticoagulant rivaroxaban.

Several current randomized controlled trials (RCTs) are testing antithrombotic treatments in COVID-19 patients which includes antiplatelet agents, anticoagulants, fibrinolytic agents, or combinations of these agents. At ClinicalTrials.gov, 76 studies of antithrombotic drugs for COVID-19 patients have been registered [16]. In clinical trials databases, 25 interventional trials are registered with 15 related to the use of LMWH among which six studies have been completed and the rest are ongoing [17]. 11 RCTs of antithrombotic treatment in outpatients with COVID-19 have been reported, with enoxaparin, DOACs, aspirin, and sulodexide being studied [18].

The study of the COVID-19 cases in this case series is the first kind in Dhulikhel Hospital and thus will be evidence for future researchers and physicians. This case series would have been more comprehensive and diverse, integrating cases from other institutions. This would have provided more generalized findings. Further detailing of the cases would have provided more information to the readers. Future researchers may explore improving the study design considering the limitation of the design, for precise generalization.

## 4 Conclusion

VTE following COVID-19 infection has led to the formation of many guidelines that recommend the use of a therapeutic dose of anticoagulants in hospitalized patients with COVID-19 without contraindications. Based on this, we used therapeutic doses of LMWH followed by rivaroxaban which showed good efficacy with complete resolution of the thrombus during the three months follow-ups and no bleeding episodes. With the COVID-19 pandemic still evolving, further research on individualized drug use is advisable for further risk stratification and preventing complications.

## Ethical approval

We have obtained written informed consent from the patients for the publication of this case series.

## Sources of funding

None.

## Consent

Written informed consent was obtained from the patient for publication of this case report and accompanying images. A copy of the written consent is available for review by the Editor-in-Chief of this journal on request.

## Registration of research studies

N/A.

## Patient perspective

After explaining to the patient about the entire project and its benefit for clinicians and patients globally, the patients were extremely happy.

## Author contribution

PS: Patient care, concept, manuscript writing, editing, review, guarantor; AT: Concept, Manuscript writing, editing and review; RBS: Manuscript writing and editing; SB: Manuscript writing and editing; YS: Manuscript writing and editing; RMK: Management of the case; SV: Management of the case; SB: Concept, Manuscript writing, editing and review.

## Guarantor

Dr Prakash Sapkota.

## Provenance and peer review

Not commissioned, externally peer reviewed.

## Declaration of competing interest

None.
